# Sedation of children for auditory brainstem response using ketamine-midazolam-atropine combination – a retrospective analysis

**DOI:** 10.1186/2193-1801-2-178

**Published:** 2013-04-22

**Authors:** Tímea Bocskai, Adrienne Németh, Lajos Bogár, József Pytel

**Affiliations:** 1 Department of Anesthesiology and Intensive Care Medicine, University of Pécs, 13. Ifjúság Street, Pécs, 7624 Hungary; 2 Department of Oto-rhino-laryngology and Neck Surgery, University of Pécs, Pécs, Hungary

**Keywords:** Sedation, Children, Ketamine, Intramuscular

## Abstract

Authors investigated sedation quality in children for auditory brainstem response testing. Two-hundred and seventy-six sedation procedures were retrospectively analyzed using recorded data focusing on efficacy of sedation and complications. Intramuscular ketamine-midazolam-atropine combination was administered on sedation preceded by narcotic suppository as pre-medication. On using the combination vital parameters remained within normal range, the complication rate was minimal. Pulse rate, arterial blood pressure and pulse oxymetry readings were stable, hypoventilation developed in 4, apnoea in none of the cases, post-sedation agitation occurred in 3 and nausea and/or vomiting in 2 cases. Repeated administration of narcotic agent was necessary in a single case only. Our practice is suitable for the sedation assisting hearing examinations in children. It has no influence on the auditory brainstem testing, the conditions necessary for the test can be met entirely with minimal side-effects. Our practice provides a more lasting sedation time in children during the examination hence there is no need for the repetition of the narcotics.

## Introduction

Auditory brainstem response testing helps detect hearing deficiencies in early childhood. One of the most common and most reliable auditory brainstem response testing method is BERA (Brainstem Electric Response Audiometry). At the Department of Oto-rhino-laryngology University of Pécs, Hungary BERA examinations commenced in 1979, its local development and implementation was initiated and facilitated by Bauer, Pytel and Kellényi (Pytel 1996; Pytel et al. 1982).

Other methods involved in objective audiometry testing are TEOAE (Transiently Evoked Otoacoustic Emission), DPOAE (Distortion Product Otoacoustic Emission) and MLR (Middle Latency Response).

A precise and adequate examination requires the patient to lie still which in case of children is often hard to achieve. While in case of babies it can be performed during spontaneous sleep, older children can be told not to move during the examination. However, one age group still needs sedation for relaxed circumstances at hearing examinations. The aim of the sedation of infants and small children is to make the child as tranquil and still. Relaxation and artificial respiration are not recommended, because they influence the examination, the sedation with continuous spontaneous breathing must be sustained (Akin et al. 2005).

Our aim was to study the characteristics and efficacy of the sedation method applied in children during the measurement of auditory brainstem responses in our institution back for nearly 20 years time retrospectively.

## Methods

Between 1992 and 2011 17,208 BERA examinations were performed out of which in 276 children was necessary the sedation. The youngest patient sedated was 1.5 months old the oldest was 8 years old. Hearing examinations were also performed without sedation during spontaneous sleep (e.g. after breastfeeding). At the Department of Oto-rhino-laryngology, University of Pécs, it was the first anaesthesiologist L. Somodi who developed the basic principles of the sedation of children. Based on the suggestions of the first author (T. Bocskai) certain changes were made in our protocol.

To induce sedation authors used a combination of ketamine-midazolam-atropine in the form of intramuscular narcosis (Pruitt et al. 1995; Auletta et al. 1999; Pruitt et al. 2006; Roback et al. 2006). Ketamine (Wathen et al. 2000), due to it narcotic effect, is well-applicable for inducing short-term narcosis and sedation with sustained spontaneous breathing. In order to decrease side-effects of ketamine the combination contains midazolam and atropine as well.

Midazolam (Sener et al. 2011) reduces its hallucinogenic effect and potential agitation, atropine (Heinz et al. 2006) serves to alleviate parasympathycotony. The sustention of spontaneous breathing and the avoidance of mechanical ventilation is vital during examinations. The reason for this is that as an effect of gases used during intra-tracheal narcosis pressure changes occur that alter measurement results (Guven et al. 2006). When using ketamine, striated muscle tone is kept thereby non-instrumental securing of airways is possible. Swallowing is normal. Laryngeal and pharyngeal reflexes are undisturbed.

Sedation of children is performed by the intramuscular administration of a combination of anaesthetic agents calculated according to body weight. The combination contains: ketamine (5 mg/kg) (Green et al. 1999), midazolam (0.1 mg/ kg) and atropine (0.01 mg/ kg) (Green et al. 2010; Brown et al. 2008). Anaesthetic agent is administered by the anaesthesiologist into the thigh muscle of the child. The child falls asleep within 1–1.5 minutes, spontaneous breathing is maintained. Mayo tube is not routinely used due to a potential to increase the laryngo-pharyngeal reflexes. The patient is made to lie down on the examination table in the supine position, instruments of patient monitoring and electrodes needed for the hearing examination are attached. Venous access is achieved by a 22G canul. Vital parameters (SatO_2_, pulse rate, blood pressure, ECG, breathing parameters) are continuously monitored and registered (Akin et al. 2005). After the examination until the child is fully awake he/she is taken to the postoperative care unit for continuous observation. Waking time is usually 45–60 minutes and parents are allowed to be present on waking. Children are allowed to drink short time after they are completely awake and leave the department after 2–3 hours.

Clinical admission of children occurs one day before the examination assigned. On that day oto-rhino-laryngological examinations, preoperative anesthetic assessment is performed, the patient and/or parent(s) are informed about the procedure and asked to sign the informed consent. Patients may also suffer from other chronic conditions (epilepsy, cardio-vascular or pulmonary diseases, disturbances of thyroid function, increased intracranial pressure). Having information about and controlling these preoperatively is therefore crucial. The psychological and psychiatric state of such children can often differ from normal, for this reason it is even more essential that hearing examination cause the least trauma possible. One day before the objective audiometrical testing we try to perform the measurement without anaesthesia. If it is unsuccessful the next day we can perform the examination with anaesthesia.

After anaesthesiology ward-round children with empty stomach receive pre-medication, a narcotic suppository, according to their body weight (narcotic suppositories are prepared by the University Pharmacy, there are 5 types according to drug-content, Table 1). After about half an hour children are taken to the audiology laboratory accompanied by parents.

**Table 1:** **Composition of premedication suppository**

Types	Children' bodyweight (kg)	Midazolam (mg)	Atropine (mg)
Suppository I	3-5	1	0.1
Suppository II	5-10	2	0.2
Suppository III	10-15	3	0.3
Suppository IV	15-20	4	0.4
Suppository V	20-30	5	0.5

The audiology examination room is not the usual anaesthesiological facility, children are not sedated in the operation theatre. For patient safety equipments and conditions of narcosis are all available in the examination room (patient monitoring devices, oxygen and suction pump lines and instruments for securing airways, emergency care medications and a respirator). Personnel requirements: an anaesthesiologist and nurse specialised in anaesthesiology, well aware of the physiology and pathology of babies and infants, experienced in performing the given methods and who and, if complications arise, are able to recognise and manage them (Mikos & Velkey 2004).

In the first few years children did not receive premedication. They gave signs of waking, started moving therefore the administration of narcotic agent had to be repeated (the dose is half of the initial one). The narcotic suppository contains midazolam and atropine (narcotic suppository I-IV) calculated according to bodyweight. Since the introduction of a routine-like premedication before sedation the repeated administration of narcotic agent has not been needed. It also had a positive effect on postoperative time, children woke up faster (Beebe et al, 1992).

Apart from summarizing clinical experiences, we also performed Mann–Whitney U-test statistical analysis of ketamine administration with respect to examination time, the children’s age and the use of premedication suppository. We distinguished three groups on the basis of premedication: Group 1 did not receive premedication (2000–2002), Group 2 did but not in every case (2003–2004), and children in Group 3 were all premedicated.

## Results

At the Department of Oto-rhino-laryngology, University of Pécs we have been performing BERA examinations under anaesthesia for 30 years. The present study involved 276 patients.

Apart from BERA, microscopic ear examination, TEOAE, DPOAE and if indicated MLR measurements were also performed. Consequently, sedation time including the placement of electrodes was 45–60 minutes on average. All sedations had to be planned within this interval. Out of sedated children 254 belonged to the ASA I Group (91.5%) and 22 into the ASA II Group (8.5%). Average age was 45 months (the youngest was 1.5 months the oldest 6 years old) bodyweight on average was 12 kg (5–22 kg). Distribution of sex was unequal, out of the 276 children 182 were boys and 94 girls (Table 2).

**Table 2:** **Demographic data**

	Mean (min.-max.)
Age (months)	45 (1.5-96)
Bodyweight (kg)	12 (5-22)
Pulse rate (min^-1^)	132 (100-156)
Systolic blood pressure (mmHg)	87 (80-110)
SatO2 (%)	99 (96-100)
Sedation time (min)	31 (18-45)
Recovery time (min)	45 (10-120)
	Number
Auditory examination / sedation of children	1730 / 276
Boys / girls	182 / 94
ASA groups (I / II)	254 / 22 (91.5% / 8.5%)
Apnoe	276 / 0 (0%)
Hypopnoe	276 / 4 (1.5%)
Coughing	276 / 1 (0.4%)
PONV	276 / 2 (0.7%)
Agitation	276 / 3 (1.1%)
Antidote administration	276 / 1 (0.4%)

SatO_2_ = oxygen saturation of peripherial hemoglobin, PONV = postoperative nausea and vomiting.

During the study period ketamine was excluded from the combination on four occasions (1.48%) due to contraindication. In these cases a repeated dose of midazolam was required. Circulatory parameters observed were stable in all cases, mean systolic pressure was 87 mmHg (min. 80, max. 110) and mean pulse rate was 132/ min (min. 100, max. 156), arrhythmias or significant changes in blood pressure were not observed. Oxygen saturation did not vary considerably, it was 99% on average (96–100%). In four cases (1.5%) hypopnoea occurred (with minimal desaturation, minimum was 96%) in Groups 1 and 2 three cases, in Group 3 one case, apnoea was not observed. These we could immediately manage with the insertion of the Mayo tube, if necessary with assisted breathing via mask and oxygen supplementation for a few minutes. In all cases the breathing of the children settled rapidly, the examination and sedation proceeded in due course. We observed coughing in one case (0.37%) in the second group. One small child was given antidote (flumazenil) after sedation since he was still not awake 2 hours after. This case was one of those where we did not give ketamine only midazolam. Nausea and vomiting occurred in 2 cases (0.74%) after the examination. Waking was smooth, agitation occurred in 3 cases (1.11%). Table 2 contains vital parameters and complications too. Children drank after 45–60 minutes and left the department within a few hours. Complications within 24 hours following discharge were unknown.

The average examination time showed a slight increase (Group 1: 48 ± 13 (SD) min, Group 2: 47 ± 14 min, Group 3: 51 ± 12 min, p < 0.05). This however, is a positive result since examinations have been extended throughout the years which have lead to a longer examination time. Although, average ketamine use was higher in Group 3 (45.2 ± 15.6 mg) than in the other 2 groups (Group 1: 36.9 ± 13.1 mg, Group 2: 38.5 ± 15.4 mg, p < 0.01). The mean age of Group 3 was higher (40.8 ± 20 months) than in Group 1 and 2 (31.2 ± 14.8, 31.9 ± 15.9 months) (Table 3).

**Table 3:** **Mean age, sedation time and ketamin dose ± SD**

	Group 1	Group 2	Group 3
Age (months)	31.2 ± 14.8	31.9 ± 15.9	40.8 ± 20
Sedation time (min)*	48 ± 13.2	47 ± 14.2	51 ± 11.7*
Ketamin (mg)**	36.9 ± 13.1	38.5 ± 15.4	45.2 ± 15.6**

*=p<0.05, **=p<0.01 compared to Group 1.

As a control we calculated the average duration of one potential measurement in the children without anaesthesia (174 children). The duration depends on the noisy circumstances. In the group of children without anaesthesia the average duration of one potential measurement was 2,61 ± 1,14 min. In the group of children with anaesthesia this value was 2,5 ± 0,99 min. The difference is not significant.

## Discussion

In the practice of other institutes, for the sedation of infants and small children propofol (Purdie & Cullen 1993; Godambe et al. 2003; Miner et al. 2010) a combination of propofol-ketamine (Sharieff et al. 2007) are administered intravenously or chloralhydrate (Buck 2005; Rumm et al. 1990) per os. As compared to ketamine the use of propofol may lead to a higher incidence of hypopnoea-apnoea and low blood-pressure, however, nausea-vomiting upon waking is rare.

Pain can be common on administration. As side-effects of chloralhydrate cardio-respiratory depression, arrhythmias, vomiting and hyperbilirubinaemy may occur. Its half-time is long (9–40 hours) therefore with repeated use there is an increased risk of accumulation and toxicity.

In our department the examination is in the frame of “one day surgery” protocol, the duration is within 24 hours, therefore, extended waking times (Akin et al. 2005; Roback et al. 2006; Godambe et al. 2003; Krauss & Green 2000) and the postoperative observation of the patients at postoperative care units does not cause staffing or organisation problems. Examinations are scheduled on less busy surgical days so that children can wake up under peaceful circumstances at postoperative care units.

Ketamine used in combination proved an adequate basis in the sedation of children (Buck 2005; Rumm et al. 1990; Krauss & Green 2000). Research suggests that intramuscular ketamine administration has more advantages than intravenous (Pruitt et al. 2006; Roback et al. 2006). Supplementing ketamine with midazolam and atropine with the aim to reduce side-effects proved to be effective. It does not cause anti-cholinergic side effect and agitation is rare (3 cases, 1.1%). Circulatory parameters were stable in all cases, blood pressure and pulse rate remained within the normal range for the children’s age. Research data of (Krauss & Green 2000), (Sury et al. 2010) and (Ng & Ang 2002) support the efficacy of ketamine and the favourable conditions of sedation it provides. Spontaneous breathing was sustained in all cases, breathing depression occurred in 4 cases (1.5%), apnoea did not occur in our group. Mean oxygen saturation was 99%, the minimum 96%. Nausea and vomiting after examination occurred with 2 children (0.7%). (Akin et al. 2005) had similar findings on sedations for BERA examinations. They used and compared sedations with propofol and propofol-ketamine. In their summary they mentioned similar results the incidence of respiratory complications (oxygen desaturation, apnoea did not occur), PONV (nausea-vomiting appeared in 2 cases) and agitation (due to benzodiazepine supplementation did not occur) were low. (Godambe et al. 2003) compared sedations with propofol/fentanyl and ketamine/midazolam and similarly described ketamine as having a smaller depressive effect on breathing. (Green et al. 1998) detected respiratory complications in 1.4% and agitation in 1.6% of cases when administering intramuscular ketamine. (Miner et al. 2010) in a randomised trial where they used ketamine in adults described subclinical breathing depression which they defined on the basis of changes in ETCO_2_ and oxygen saturation. When using ketamine its frequency was 60% (30/47), when using propofol it was 40% (20/50).

We can draw the following conclusions from the analyzed statistical data. Average examination time increased due to the fact that further examinations had to be included in the procedure of the hearing examination. Still, the narcotic dose did not have to be repeated in Group 3, which is an important benefit. The frequency of occurring side-effects is smaller in Group 3. Even when a single dose of narcotic is administered, individual differences in the side-effects can occur. We have not found signification in the correlation of the administered narcotic dose, examination time and age (results is not indicated). The weight of children can vary greatly at this age group, so the narcotic administered to the child is not in correlation with age. We need to emphasize the fact that in Group 3 narcotic dose did not have to be repeated and certain side-effects were scarce. Our investigation shows that intramuscular narcotisation can be applied in the anaesthetic practice based on proper indication. The narcosis used in auditory brainstem response testing requires a sedation practice with the fewest possible side-effects. Narcosis achieved by intramuscular injection entails a trauma not greater than a venous access; on the contrary it can be done faster and more easily. During the lengthy time investigated no side-effects has occurred owing to the intramuscular access since the introduction of own practice many years ago. Children assigned for hearing examinations may suffer from various psychological conditions of hearing impairment, which makes communication more difficult. For this reason, a fast and reliable sedation practice is crucial. This way the circumstances for sedation are reassuring for parents, patients and doctors, as well.

In conclusion, we can say that adequate conditions can be provided by the intramuscular ketamine-midazolam-atropine combination used in our study for hearing examinations of infants and small children by observing minimum requirements of anaesthesia. The observation of children was continuous with the monitoring of vital parameters during the examination and the time spent in postoperative care as well. Sedation is well introducible into the examination procedure. Curves registered under the motion-free, relaxed circumstances proved more artefact-free than awake ones. Based on recent experiences the protocol we use is fully reliable and well-controllable. The combination of medication we used provided stable vital parameters throughout, complications were minimal. The routine application of the premedication suppository decreased ketamine requirement of sedation.

Intramuscular injection of children is a painful and very distressing procedure. It is blunted by the premedication but in the future a topical analgetic will be required to exclude this disadvantageous component of our methods.
